# Adaptive Copy Number Evolution in Malaria Parasites

**DOI:** 10.1371/journal.pgen.1000243

**Published:** 2008-10-31

**Authors:** Shalini Nair, Becky Miller, Marion Barends, Anchalee Jaidee, Jigar Patel, Mayfong Mayxay, Paul Newton, François Nosten, Michael T. Ferdig, Tim J. C. Anderson

**Affiliations:** 1 Southwest Foundation for Biomedical Research (SFBR), San Antonio, Texas, United States of America; 2 Department of Biological Sciences, Eck Institute for Global Health, University of Notre Dame, Notre Dame, Indiana, United States of America; 3 Shoklo Malaria Research Unit (SMRU), Mae Sot, Tak, Thailand; 4 Faculty of Tropical Medicine, Mahidol University, Bangkok, Thailand; 5 Roche NimbleGen, Inc., Madison, Wisconsin, United States of America; 6 Wellcome Trust–Mahosot Hospital–Oxford Tropical Medicine Research Collaboration, Mahosot Hospital, Vientiane, Lao People's Democratic Republic; 7 Department of Post Graduates and Research, Faculty of Medical Science, National University of Laos, Vientiane, Lao People's Democratic Republic; 8 Centre for Tropical Medicine and Vaccinology, Churchill Hospital, Oxford, United Kingdom; University of Chicago, United States of America

## Abstract

Copy number polymorphism (CNP) is ubiquitous in eukaryotic genomes, but the degree to which this reflects the action of positive selection is poorly understood. The first gene in the *Plasmodium* folate biosynthesis pathway, GTP-cyclohydrolase I (*gch1*), shows extensive CNP. We provide compelling evidence that *gch1* CNP is an adaptive consequence of selection by antifolate drugs, which target enzymes downstream in this pathway. (1) We compared *gch1* CNP in parasites from Thailand (strong historical antifolate selection) with those from neighboring Laos (weak antifolate selection). Two percent of chromosomes had amplified copy number in Laos, while 72% carried multiple (2–11) copies in Thailand, and differentiation exceeded that observed at 73 synonymous SNPs. (2) We found five amplicon types containing one to greater than six genes and spanning 1 to >11 kb, consistent with parallel evolution and strong selection for this gene amplification. *gch1* was the only gene occurring in all amplicons suggesting that this locus is the target of selection. (3) We observed reduced microsatellite variation and increased linkage disequilibrium (LD) in a 900-kb region flanking *gch1* in parasites from Thailand, consistent with rapid recent spread of chromosomes carrying multiple copies of *gch1*. (4) We found that parasites bearing *dhfr*-*164L*, which causes high-level resistance to antifolate drugs, carry significantly (*p* = 0.00003) higher copy numbers of *gch1* than parasites bearing 164I, indicating functional association between genes located on different chromosomes but linked in the same biochemical pathway. These results demonstrate that CNP at *gch1* is adaptive and the associations with *dhfr-164L* strongly suggest a compensatory function. More generally, these data demonstrate how selection affects multiple enzymes in a single biochemical pathway, and suggest that investigation of structural variation may provide a fast-track to locating genes underlying adaptation.

## Author Summary

Recent comparative genomic hybridization studies have revealed extensive copy number variation in eukaryotic genomes. The first gene in the *Plasmodium* folate biosynthesis pathway, GTP-cyclohydrolase I (*gch1*), shows extensive copy number polymorphism (CNP). We provide compelling evidence that *gch1* CNP is adaptive and most likely results from selection by antifolate drugs, which target enzymes downstream in this pathway. *Gch1* CNP shows extreme geographical differentiation; hitchhiking reduces diversity and increases LD in flanking sequence, indicating recent rapid spread within Thailand, while amplicon structure reveals multiple origins and parallel evolution. Furthermore, strong association between elevated copy number and a critical mutation *dhfr-I164L* that underlies high-level antifolate resistance indicates functional linkage and fitness epistasis between genes on different chromosomes. These data reveal hidden complexity in the evolutionary response to antifolate treatment and demonstrate that analysis of structural variation can provide a fast-track to locating genes that underlie adaptation.

## Introduction

A spate of studies over the past five years have described widespread copy number variation (CNP) within the genomes of humans, mice, *Drosophila* and other eukaryotes [1]–[5]. The existence of large regions of the genome that vary in copy number between individuals has lead to a reconsideration of the importance of structural variation for our understanding of genetic and phenotypic variation [6]. However, it is unclear whether CNP evolution is predominantly neutral, or whether positive or negative selection play significant roles in shaping the patterns observed [1]. The fact that CNPs tend to be enriched for particular gene classes, and for genes showing evidence for positive selection at the nucleotide level, strongly suggests the action of positive selection [7], although the alternative explanation of purifying selection against CNP in particular gene classes cannot be discounted. Furthermore, that CNPs explain ∼20% of variance in transcript abundance in humans suggests that they have the potential to make a significant contribution to disease susceptibility and adaptive evolution [8]. However, despite these indirect lines of evidence for positive selection, adaptive copy number evolution has been demonstrated or hypothesized in only a few cases. In humans there are two notable examples. Gonzales et al [9] showed that protection from HIV is associated with CNP at the CCL3L1 gene. This CNP shows extreme geographical variation which further supports the action of selection by HIV (or, more likely, by an older human pathogen) [10]. Similarly, Perry et al [11] showed higher copy number of the amylase gene in populations with high starch diets.

CNP is also widespread in the malaria parasite genome [12],[13]. Malaria parasites are exposed to strong selection from the human immune response and treatment with antimalarial drugs. They have relatively small genomes (23 Mb) and haploid genetics, and can be grown and genetically manipulated in the laboratory, so provide a useful eukaryotic organism for investigating the functional role of CNP. One CNP on chromosome (chr.) 5 is known to underlie a multidrug resistance phenotype: chromosomes carrying this CNP have risen to high frequencies in Southeast Asia [14],[15] and manipulation of copy number alters response to multiple drugs [16]. However, this one example of adaptive copy number variation in *P. falciparum* has been regarded as an exceptional case, and SNP based approaches have been prioritized as the primary tool for mapping functional genes in *Plasmodium* [17].

**Figure 1:**
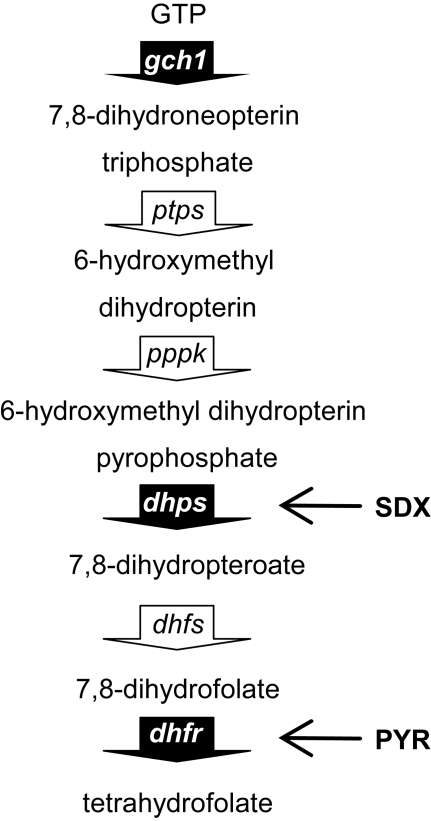
The folate biosynthesis pathway of *P. falciparum*. The steps catalyzed by *gch1*, *dhfr* and *dhps* are highlighted. The positions at which antifolate drugs (Pyrimethamine (PYR) and Sulfadoxine (SDX)) target the pathway are marked. Abbreviations: pyruvoyltetrahydropterin synthase (*ptps*), hydroxymethyldihydropterin pyrophosphokinase (*pppk*), dihydrofolate synthase (*dhfs*). Modified from [18].

The first cGH study of *P. falciparum* in 16 laboratory isolates revealed a particularly interesting CNP containing GTP-cyclohydrolase I (*gch1*) [12]. This gene encodes the first and rate limiting enzyme in the folate metabolism pathway (Figure 1) [18],[19]. Two key enzymes in later stages of this pathway–dihydrofolate reductase (*dhfr*) (chr. 4) and dihydropteroate synthase (*dhps*) (chr. 8)–are targets of the antifolate drugs pyrimethamine and sulfadoxine, which are combined in the drug Fansidar (Roche). This drug replaced chloroquine as the first-line treatment against malaria in many countries, but resistance has spread rapidly where it has been deployed. Specific point mutations (N51I, C59R, S108N, I164L) in parasite *dhfr* alter the binding of pyrimethamine to the enzyme's active site [20]. In addition to causing resistance, mutations in *dhfr* reduce enzyme efficacy and carry adverse fitness effects [21],[22],[but see 23]. Similarly, mutations in *dhps* (S436A/F, A437G, K540E, A581G, and A613T/S) underlie resistance to sulfadoxine [20]. Kidgell et al [12] speculated that increased gene dosage might play a compensatory role in antifolate resistance by increasing flux in the pathway to compensate for reduced efficacy of *dhfr* and/or *dhps* genes bearing resistance mutations.

This study was designed to determine whether CNP at *gch1* is a consequence of adaptive evolution. To do this, we examined the population genetics of *gch1* CNP in Laos and Thailand. These two neighboring countries in SE Asia have contrasting selection histories with antifolate drugs. In Western Thailand antifolate drugs were the first line treatment from 1970–80, and resistance mutations at both *dhps* and *dhfr* are fixed or at high frequency [24],[25]. By contrast, in Laos antifolate drugs were the second line drug until 2006, but were seldom used [26], and resistance mutations are at lower frequencies in both *dhfr* and *dhps* [25],[27]. We provide compelling evidence–from patterns of population differentiation, hitchhiking, and amplicon structure–that the *gch1* CNP (chr. 12) in *P. falciparum* results from recent adaptive evolution that is most likely associated with antifolate treatment. Furthermore, we show strong association between *gch1* CNP and a critical mutation in *dhfr*, indicating functional linkage between genes on different chromosomes in the same biochemical pathway.

## Materials and Methods

### Sample Collection

We collected 5 mL blood samples from *P. falciparum* infected patients visiting the malaria clinic at Mawker-Thai on the Thai-Burma border between December 2000 and September 2003. These patients had not visited the malaria clinic within the past 60 days, and had no history of prior malaria treatment during this time. The clinic serves people on both sides of the border; 90% of patients travel from within a 10 km radius around the clinic (François Nosten, personal communication). We collected samples from Phalanxay (Savannaket) in Laos between June 2002 and Sept 2003 from patients involved in clinical drug efficacy trials. Collection protocols were approved by the Ethical Committee of the Faculty of Tropical Medicine, Mahidol University, Bangkok, and by the Institutional Review Board at the University of Texas Health Science Center at San Antonio. Parasite DNA was prepared by phenol/chloroform extraction of whole blood, following removal of buffy coats as described previously [28].

### Plasmodium Genetics and Biology

Malaria parasites are obligately sexual protozoans. Haploid blood-stage parasites replicate asexually within the human host and differentiate into male and female gametocytes. Male and female gametes fuse in the mosquito midgut generating a short lived diploid stage (ookinete). Meiosis then gives rise to haploid infective stages (sporozoites) that are transmitted to humans during mosquito feeding. Self-fertilization predominates in regions of low transmission such as Thailand and Laos, because most infected humans, and therefore mosquito blood meals, contain a single parasite clone. The recombination rate is high (1 cM = 17 kb), but the effective recombination rate is modified by the rate of selfing in natural parasite populations. The *effective* rate of recombination (*r′*) is given by *r′ = r(1-F)* where *r* is the recombination rate and *F* is the inbreeding coefficient [29],[30]. In Thailand the inbreeding coefficient is estimated to be between 0.6–0.9 [31]. The generation time (from mosquito to mosquito) is variable, but is estimated to be ∼8 weeks [31].

### Microsatellite Genotyping

We used microsatellite genotyping to identify infections containing >1 parasite clone. We genotyped seven di- or tri-nucleotide markers distributed on different chromosomes: ARA2 (chr. 11), POLYα (chr. 4), TA1 (chr. 6), C2M1 (chr. 2), C3M54 (chr. 3), TA60 (chr. 13), and C4M30 (chr. 4). Primers and protocols for amplifying these loci are described in [32]. We amplified microsatellites using fluorescent end-labeled oligos, ran products on an ABI 3100 capillary sequencer, and scored alleles using GENESCAN and GENOTYPER software. Multiple clone infections were defined as those in which one or more of the seven loci showed multiple alleles. Minor alleles were scored if they were >33% the height of predominant alleles [33].

We genotyped 33 dinucleotide microsatellites on chr. 12 flanking *gch1* to examine expected heterozygosity and LD. These included 10 markers situated within a 14 kb window containing *gch1*. Oligos and locus details are listed in Table S1. These were genotyped using the methods described above. However, for 17/33 loci we used the M13 tailing method [34] in which the fluorescent labeled M13 oligo (tgtaaaacgacggccagt) was added to the 5′ of the forward oligo to label PCR products.

### Measurement of *gch1* Copy Number and Characterization of Amplicons

We used a real-time PCR assay to measure copy number of *gch1* relative to single copy gene (PFL0770w: Seryl-T synthetase). Assay details are provided in Table S2. We measured amounts of *gch1* or Seryl-T synthetase PCR products using Minor Groove Binding (MGB) probes (Applied Biosystems, Foster City, CA). We used the ΔΔC_T_ method to measure copy number relative to a standard calibrator sample. We initially used the sequenced parasite strain 3D7 as the calibrator. However, initial trials indicated that this parasite line has multiple copies of *gch1*, as observed previously [12]. We therefore measured gene copy number relative to a field sample with a single copy of *gch1*. All assays were run in quadruplicate in 10 µl volumes on 384-well plates on an ABI 7900HT real-time PCR machine. We excluded real-time PCR data when the C_T_ was >32 for either test or reference gene, or if the upper 95% confidence interval around the copy number estimate is >0.4, or if the estimated copy number was <0.5. For some analyses the continuous real-time estimates were made discrete, by rounding to the nearest integer value. To ensure data comparability we run real-time assays for samples from Thailand and Laos on the same 384-well plates and included parasite 3D7 (which had 4.5±0.36 (1 s.d.) copies in 8 replicate assays) as a positive control on each plate.

We also used real time assays for genes flanking *gch1* on chr. 12 to identify the size and gene content amplified chromosomal regions (table S2) while we amplified and sequenced breakpoint specific PCR products (table S3) to identify the precise position of the chromosomal breakage points (Figure S1) using methods previously described [14].

### *Gch1* Sequencing

We sequenced gch1 from 24 parasites with a single copy of gch1 from Laos and from 24 parasites from Thailand (8 with single copies and 16 with multiple copies). 1205 bp sequences were PCR amplified using the oligos TTCATTTAATGGACTGGAAA and GGCTAATTTAAATTTTCCAC. Amplified products were sequenced directly on both strands using the BigDye Terminator v3.1 cycle sequencing kit (Applied Biosystems, Inc., Foster City, CA) and internal oligos CATTACTTTTATTTCCTTCC, CATCTTTTACCTTTTGAAGG, GAACAAATAGAAGATATGCTG, and CTTCAAAAGGTAAAAGATGG. BigDye products were cleaned using Sephadex G-50 (Sigma-Aldrich Co., St. Louis, MO) prior to sequencing.

### SNP Genotyping

We used primer extension to determine point mutations in *dhfr* and *dhps* associated with antifolate resistance in *P. falciparum* using protocols described previously [28]. Genotyping was performed by primer extension using the ABI PRISM SNaPshot Multiplex Kit (Applied Biosystems) and the products of the SNaPshot reactions were scored on an ABI 3100 capillary sequencer using GENESCAN and GENOTYPER software. This method allows simultaneous genotyping of all five mutations in *dhfr* or *dhps* in single reactions [28].

We used the Illumina BeadXpress platform to genotype 96 synonymous SNPs. Targeted SNPs were chosen using the query system in www.plasmodb.org. We searched for synonymous sites that were polymorphic in the genome sequence data from laboratory parasites Dd2, FCC-2, FCB, K1, VI/S. These were selected from genes showing low levels of variation and dN/dS ratios <1. We also excluded surface expressed genes and transporters from our searches, as well as genes in telomeric regions (100 kb from chromosome ends) because these are enriched for antigenic loci. Only SNPs that were deemed designable by Illumina (with SNP scores>0.4) were included (table S4). Genotyping was carried out according to the Illumina BeadXpress instructions, except we used 25 ng DNA rather than 250 ng starting DNA. We included parasites FCC-2, FCB, K1, VI/S as controls. Two SNPs were not scoreable, while 4 others gave high levels (>5%) of missing data. Five loci were monomorphic in the controls suggesting that they result from errors in the existing genome sequence data. Of the remaining 85 loci, 74 (87%) were polymorphic in at least one sample, while 73 had minor allele frequencies >1% in at least one of the two populations examined.

### Parasite Culture and Expression Analysis

We compared *gch1* gene expression in 6 parasite isolates that varied in *gch1* copy number, to determine if expression is a reflection of gene dosage. Parasites were cultured at 90% N_2_, 5% Co_2_ 5% O_2_ in complete media containing albumax, synchronized using sorbitol, and aliquots were preserved in TriReagent (Molecular Research Center Inc.) for RNA extraction. We treated samples with DNase 1(Invitrogen) to ensure no genomic DNA was present and confirmed absence of DNA by PCR. mRNA was transcribed using RNA PCR core kit (Applied Biosystems), to generate cDNA. We quantified levels of cDNA with the Taqman assay run on the ABI PRISM 7900 using the oligos listed in Table S2.

### Statistical Analysis

We used t-tests and Mann-Whitney U-tests to investigate association between SNPs and CNP at *gch1*. Copy number was log transformed for parametric analyses. We measured expected heterozygosity (*H_e_*) at each locus using the formula *H_e_* = [*n*/(*n*−1)][1−Σ*p_i_* ^2^], where *n* is the number of infections sampled and *p_i_* is the frequency of the *i*th allele. For haploid blood stage malaria parasites this statistic measures the probability of drawing two different alleles from a population. The sampling variance of *H_e_* was calculated as *2(n−1)/n^3^{2(n−2)[*Σ*p_i_^3^−(*Σ*p_i_^2^)^2^]}* [a slight modification of the standard diploid variance [35]]. To examine patterns of LD surrounding *gch1* we measured Extended Haplotype Homozygosity (*EHH*) [36] where *EHH* at a distance *x* from the gene of interest is defined as the probability that two randomly chosen haplotypes are homozygous for all microsatellites in this region. These statistics (±1 SD) were measured using the *EHH* Calculator [37]. We calculated *F_ST_* following [38] using FSTAT2.9.3 [39].

## Results/Discussion

### Extensive CNP at *gch1* in Thailand

**Figure 2:**
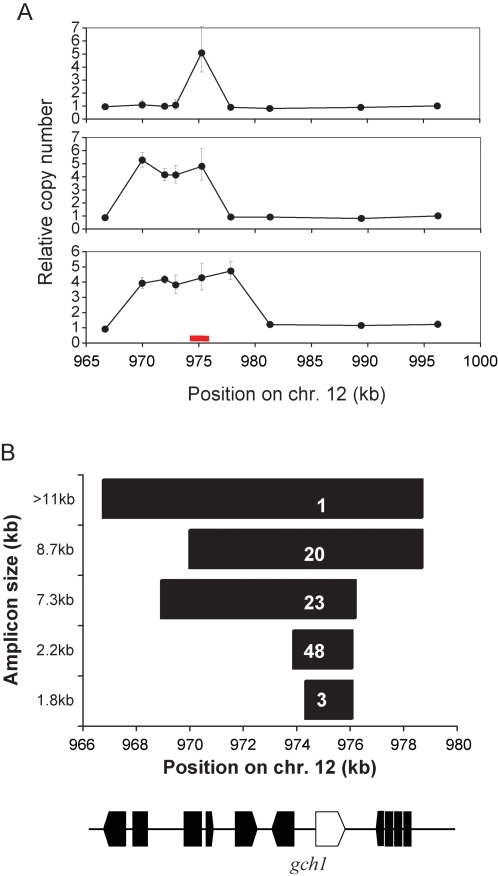
Structure and gene content of *gch1* amplicons. The span of each amplicon was established by real-time PCR and breakpoint specific PCR assays. (a) Real-time PCR estimates of relative copy number plotted for 9 genes including gch1 on chr. 12. The error bars show 95% confidence intervals around the copy number estimate. The three plots show the profiles observed for the 2.3 kb (top), 7.3 kb (middle) and 8.7 kb (bottom) amplicons. The location of *gch1* is marked by a red bar (b) Plot showing the span of the 5 amplicons types. The abundance of each of the amplicon types in the Thai sample is shown in white on the bars. The gene content in this region of the chr. 12 are shown beneath the graph: all breakpoints were between genes. The 5′ boundary for the >11 kb amplicon fell outside the range of our real-time PCR assays and was not defined: the bar shows the minimum size estimate for this amplicon.

Initially, we sampled *P. falciparum* from infected patients visiting a single clinic on the Thailand-Burma border. Following removal of multiple clone infections 140 samples were available. We found between 1 and 11 copies of *gch1* by taqman PCR with 72% of parasites sampled carrying >1 copy. To determine the arrangement and size of these genome amplifications we measured copy number in genes surrounding *gch1* (Figure 2a) and designed PCR assays to identify chromosome breakpoints [14] (Table S3, Figure S1). We found 5 different amplicon types containing one to >six genes (Figure 2b). Breakpoint specific PCR assays indicate that these were arranged in tandem. However we note that duplicative transposition of some amplicons would not be detectable using this assay. The most common of these amplicons contained only *gch1* and accounted for 48% of all amplicon types, while the largest amplicon (>11 kb), was found in only one sample. We sequenced the chromosome breakpoints for 4 of the 5 amplicons. In each case breakpoints were found in between genes in microsatellite sequences or monomeric tracts (Figure S1). The amplicon size data provides a natural mapping experiment. *Gch1* was the only gene observed in all amplicons. Hence, if CNP results from selection, then *gch1* is clearly the gene that is targeted.

### *Gch1* CNP Shows Extreme Geographical Variation

**Figure 3:**
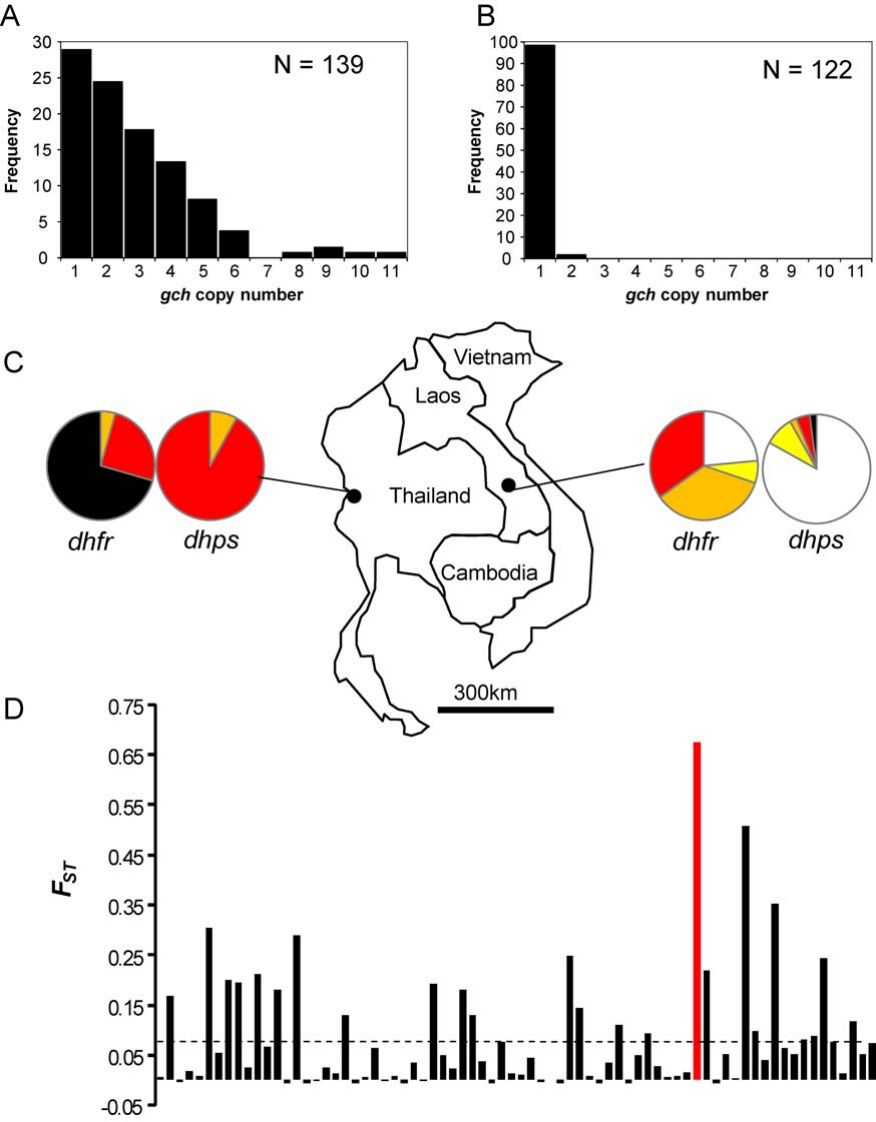
Geographical differentiation of the *gch1* CNP. The frequency of chromosomes carrying different *gch1* copy number is plotted in Thailand (a) and Laos (b). (c) Map showing sampling locations in Thailand and Laos. The pie charts show the representation of alleles present at known genes involved in antifolate resistance, and provides a molecular indicator of the strength of antifolate selection on these two populations. White-wild type, yellow-1 mutation, orange-2 mutations, red-3 mutations, black-4 mutations. See table S5 for details of the alleles present. (d) The distribution of *F_ST_* for *gch1* CNP and for 73 polymorphic sSNPs. The markers are plotted by position across the genome. The mean *F_ST_* for sSNPs is marked by the dotted line.

Comparing patterns of geographical differentiation in neutral and putatively selected loci provides a powerful approach to identify loci that underlie adaptation [25],[40],[41]. This approach is based on the premise that allele distribution at neutrally evolving loci will be determined by mutation and drift alone, while selection will influence patterns observed at loci involved in adaptation. We measured *gch1* copy number in parasites from Phalanxay (Southern Laos) for comparison with Thailand. These neighboring countries differ considerably in history of antifolate treatment [25],[27]. In Thailand, there was intensive selection with antifolates from 1970–1980 [42], whereas in Laos antifolates were the official second line treatment for malaria until 2006, but in reality they were very seldom used [26]. Differences in treatment policies and antifolate selection are evident from patterns of polymorphism at *dhfr* and *dhps*. All parasite samples (n = 139) examined from Thailand carried between 2 and 4 mutations at *dhfr*, and 80% of parasite isolates carried the *dhfr*-164*L* mutation, while at *dhps* all Thai parasites carried 2 or 3 mutations conferring resistance (Figure 3, Table S5). In contrast, in Laos the *dhfr*-164L mutation was absent, and 23% of parasites carried wild type *dhfr* alleles, while 92% of parasites carried wild-type alleles at *dhps* (Figure 3, Table S5).

While 72% of Thai parasites carry >1 copy of *gch1*, in Laos we found just 2/122 (1.6%) parasites with >1 *gch1* copy (Figure 3a). Hence, there is strong differentiation (*F_ST_* = 0.67 [grouping chromosomes with single or multiple copies of *gch1*]) between parasites sampled ∼500 km apart. The real-time copy number data is supported by PCR assays of chromosome breakpoints for 4 of the 5 amplicon types (Table S3). Using these assays, we detected tandem repeats in 72% of Thai samples, but in only 1% of samples from Laos. One of the two parasites from Laos with >1 copy of *gch1* carried the 1.8 kb amplicon, while the other had the >11 kb amplicon real time PCR profile (Figure 2). To compare differentiation at this *gch1* CNP with neutral expectations, we genotyped 96 synonymous SNPs (sSNPs) situated on all 14 chromosomes (Table S4) in the same population samples. These sSNPs were located in genes with dN/dS ratios <1 and were polymorphic in genome sequence data from SE Asian parasites [43],[44]. The *gch1* CNP shows greater differentiation than all 73 polymorphic sSNPs (defined as those loci with >1% frequency of the minor allele in at least one population) consistent with strong local adaptation (Figure 3d). Caution is needed when comparing *F_ST_* at SNPs and CNPs because both the rate and the directionality of mutation are likely to be radically different. However, we suggest that comparing SNPs with CNP will provide a conservative test, because copy number mutations typically occur at higher rates than SNPs [45] and may show frequent reversions to the single copy state. Hence, homoplasy is expected to reduce geographical differentiation at CNPs. The geographical distribution of *gch1* CNP is suggestive of strong local adaptation, but on its own does not constitute proof. Furthermore, the high frequency of *gch1* CNP in Thailand is consistent with the hypothesis that amplification is driven by antifolate treatment.

### Hitchhiking Demonstrates Rapid Recent Spread of Copy Number Variants

**Figure 4:**
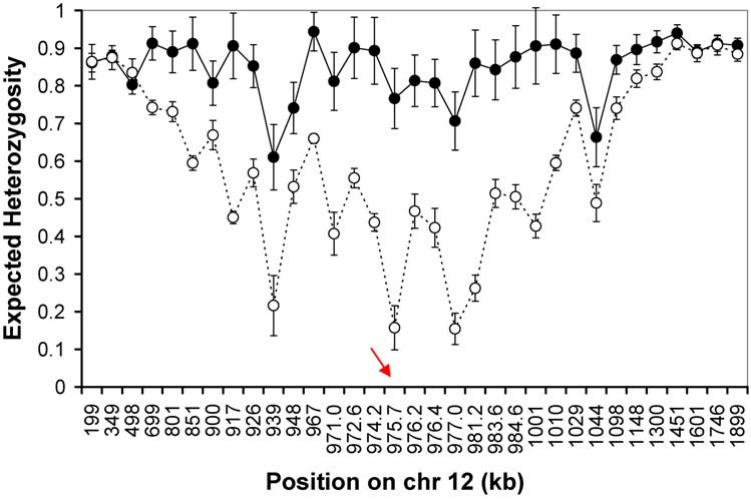
Genetic Diversity on chr. 12 in Thailand and Laos. Expected Heterozygosity plotted across chr. 12 in Thailand and Laos. The red arrow marks the position of *gch1*. Markers are ordered on the x-axis which is not shown to scale.

**Figure 5:**
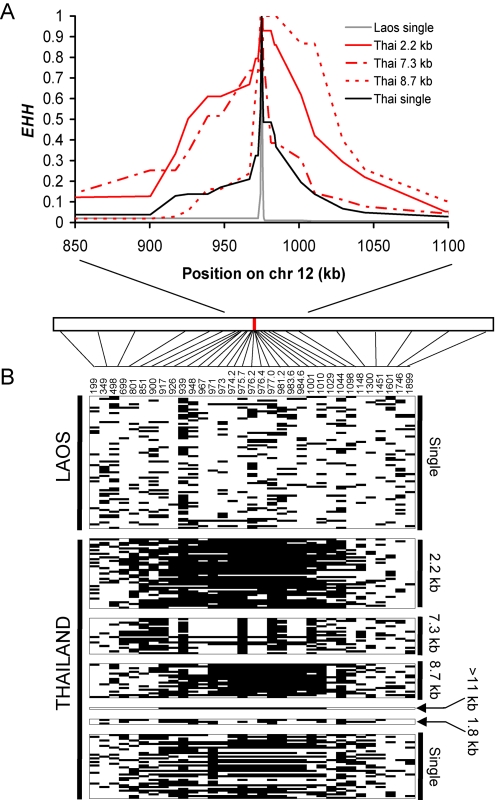
Haplotype structure and LD decay around *gch1*. (a) Plots of *EHH* for markers flanking *gch1* from Thailand and Laos. In Thailand, *EHH* is plotted for the three predominant amplicon types (2.2, 7.3 and 8.7 kb). *EHH* for chromosomes with single copies of *gch1* from both countries is shown for comparison. (b) Visual representation of LD. Microsatellite data was reduced to binary form by coloring the predominant allele in black and all other alleles in white. The 33 loci typed are arranged in order across the chromosome, while samples are arranged in rows. The country of origin of each sample is shown on the left, while the amplicon type (Figure 2) is shown on the right.

If chromosomes carrying *gch1* CNP have been under strong recent selection in Thailand, we would predict that genetic diversity would be reduced and LD increased in the vicinity of *gch1* on chr. 12. We measured length variation at 33 di-nucleotide microsatellite markers (Table S1) distributed across chr. 12 in parasites from the Thai-Burma border and Laos. These included a cluster of 11 markers within 14 kb of *gch1*. Expected heterozygosity (*H_e_*) was high across chr. 12 in Laos (mean = 0.85, s.d = 0.08) and at markers situated far from *gch1* in Thai parasites. However, for the 11 markers flanking *gch1* variation was halved in Thai parasites (*H_e_* (±s.d.) = 0.83 (±0.07) in Laos vs *H_e_* = 0.41±0.16 in Thailand) (Figure 4) and variation was reduced in Thailand for 600–950 kb (40–63 cM). These data are consistent with strong recent selection acting on *gch1* CNP. Comparison of this selective event with selective sweeps around known drug resistance genes provides a means to assess the strength of this selective event. We have previously described patterns of microsatellite variation around *dhfr* (chr. 4), the chloroquine resistance transporter (*pfcrt* (chr. 7) using parasites collected from the same clinic [27]. We observed significant reduction in variation for 98–137 kb (6–8 cM) around *dhfr*, and for 195–268 kb (11–16 cM) around *pfcrt*. The window of reduced variation is considerably larger around *gch1*, emphasizing that selection on this locus was strong and recent. Examination of extended haplotype homozygosity (*EHH*) also reveal striking differences in LD between parasites from Thailand and Laos. In Laos, haplotype blocks are short and *EHH* decays to <0.02 within 3 kb on either side of *gch1*. In contrast, haplotype blocks extend largely unbroken for 63 kb (4 cM) around *gch1* in Thai parasites with multiple copies of each of the three predominant amplicon types bearing *gch1*. These data are consistent with rapid recent spread of this CNP in Thailand (Figure 5).

### Parallel Evolution of Structural Changes

We used the amplicon sizes and microsatellite haplotypes in markers flanking *gch1* to infer evolutionary origins of these gene amplification events. Amplification of *gch1* CNP has occurred at least three times in Thailand. The 2.2 kb, 8.7 kb, and >11 kb amplicon share identical or similar backgrounds, suggesting that they share a common origin. We counted the mean proportion of shared alleles between all pairwise combinations of parasites carrying the 2.2 kb, 8.7 kb, and >11 kb amplicon for the 10 markers (12_970980–12_984557, Table S1) immediately flanking *gch1*. 94.6–98.1% of alleles were shared between chromosomes bearing these three amplicon types (Table 1). In contrast, the markers flanking the 1.7 and 7.3 kb amplicons were divergent from one another (42.0% shared alleles) and from the 2.2 and 8.7 and >11 kb amplicons (33.8–38.0% alleles shared). The simplest explanation is that the 2.2 kb, 8.7 kb, and >11 kb amplicon share the same origin, and that the 1.7 kb and 7.3 kb amplicons have evolved independently. The observation that three amplicons share the same flanking markers suggests that initially large amplicons have been progressively reduced in size. Such amplicon size reduction has been observed previously in *E. coli* [46]. In this case, deletion of genes from large amplicons results in increased growth rates and higher fitness. We expect that similar deleterious fitness effects of large amplicons may lead to progressive reduction in amplicon size in this system. Interestingly, chr. 12 bearing a single copy of *gch1* from Thailand show surprisingly strong LD compared with single copy chromosomes from Laos. Furthermore, haplotypes surrounding *gch1* on single copy chromosomes are similar to those seen in chromosomes bearing the 2.2 kb, 8.7 kb, and >11 kb amplicons (Figure 5b). These observations suggest that chromosomes carrying multiple copies of *gch1* frequently revert to single copy status. Reversion is predicted to be common in the evolution of tandem amplifications [10] and contrasts with SNPs where reversion is rare. Importantly, such reversion events will limit the power of within-population long range haplotype tests [36] to detect evidence for selection and will reduce LD between CNP and flanking SNPs reducing the power of SNP based genome wide association studies [1].

**Table 1:** Proportion of alleles shared at microsatellite loci flanking *gch1* on chromosomes bearing different amplicon types.

	Single	1.7 kb	2.2 kb	7.3 kb	8.7 kb	>11 kb
**Single**	*0.537*					
**1.7 kb**	0.438	*0.419*				
**2.2 kb**	0.734	0.387	*0.897*			
**7.3 kb**	0.467	0.417	0.380	*0.793*		
**8.7 kb**	0.699	0.367	**0.946**	0.345	*0.963*	
**>11 kb**	0.689	0.367	**0.947**	0.339	**0.981**	*1.000*

We compared haplotypes at 10 microsatellite markers in a 14 kb region containing *gch1*. Comparisons showing >90% similarity are shown in bold. Chromosomes carrying the 2.2, 8.7 and >11 kb amplicon show identical or near identical backgrounds suggesting they have a common origin. The proportion of shared alleles observed between chromosomes carrying the same amplicon type is shown on the diagonal in italics.

In the previous paragraph we argued that three of the five amplicons have a common origin and reversion to single copy status is frequent. However we cannot prove this scenario with the current data and less parsimonious alternative evolutionary scenarios are possible. For example, one haplotype could have spread to high frequency prior to copy number amplification. The three different amplicon types may have then evolved independently on the same genetic background.

We have assumed so far that CNP itself is adaptive. However, it is conceivable that CNP is linked to SNPs in *gch1*, which are the true target of selection. We therefore sequenced 48 *gch1* alleles. These sequences (940 bp) were from 24 Thai samples, representing all five amplicon types as well as single copy *gch1*, and from 24 single copy *gch1* alleles from Laos. Sequence polymorphism can be difficult to detect in samples with high *gch1* copy number. However, of 16 samples with multiple copies of *gch1* sequenced only 3 carried >4 copies, so we do not believe our ability to detect sequence variants was severely impaired. We found just one non synonymous SNP (G→T, M169I) in one each of the Thai and Lao samples, demonstrating low levels of nucleotide variation in this gene. This sequence homogeneity supports the argument that copy number, rather than associated coding SNPs, are targeted by selection.

### LD between Physically Unlinked Genes in the Folate Pathway

**Figure 6:**
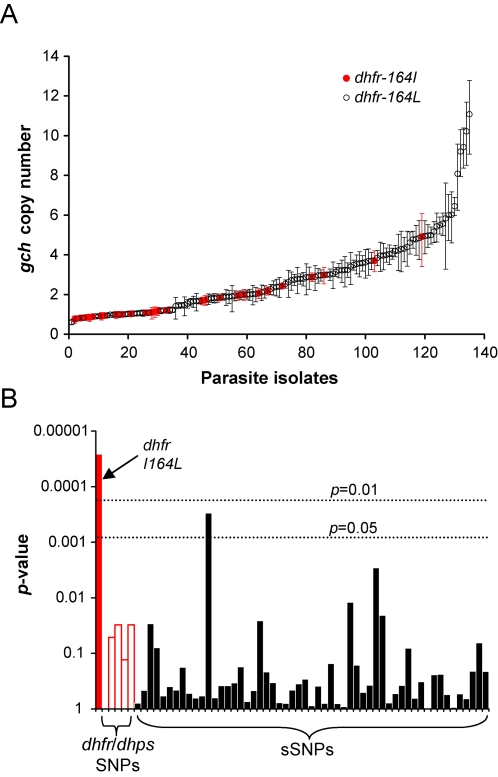
The *dhfr*-164L resistance mutation is associated with *gch1* CNP in Thailand. (a) Thai parasites were ranked by *gch1* CNP. Error bars show 95% CI around copy number estimates. Open black circles or closed red circles indicate isolates carrying 164I or 164L at *dhfr*. (b) Association between *55* polymorphic *sSNPs* and *gch1 CNP*. Raw *p*-values from t-tests of log transformed copy number estimates are plotted; very similar p-values were obtained from non-parametic Mann-Whitney U-tests using untransformed copy number estimates. Dotted horizontal lines show thresholds for significance following Bonferroni correction for multiple testing. The bars show the strength of associations between *gch1* CNP and *dhfr*-I164L (solid red), between polymorphic SNPs in *dhfr* (N51I) and *dhps* (S436A, K540E, A581G) (open red bars), and between 55 sSNPs (black). There was only one SNP (MAL04-469608) other than *dhfr-164L* that crossed the Bonferroni-corrected threshold of p<0.05.

The demonstration that a derived *gch1* CNP has rapidly spread to high frequency within Thai parasite populations, but not in neighboring populations from Laos, provides strong evidence that *gch1 is* adaptive, but provides few clues about the nature of the selection involved. We reasoned that if *gch1* CNP is involved directly or indirectly in resistance to antifolate drugs, we might expect to see genetic evidence for interactions with genes involved in resistance downstream in the folate biosynthesis pathway. We therefore examined associations between *gch1* copy number and known mutations that underlie antifolate resistance in *dhfr* (chr. 4) and *dhps* (chr. 8). In Thailand parasites bearing *dhfr*-164L carried significantly higher copy number of *gch1* (t = −4.313, p = 0.000026) than those bearing *dhfr*-164I (Figure 6a). More marginal associations were also observed for *dhfr*-N51I (t = −1.964, p = 0.051) and two sites (A436S and A581G) in *dhps* (t = −2.184, p = 0.033) in perfect LD. To empirically test the significance of the *dhfr*-164L result we compared associations between *gch1* copy number and 55 sSNPs with minor allele frequency >5% in Thailand. The *dhfr*-164L association was the strongest observed and remained significant after correction for multiple tests, arguing that this association cannot be explained by population structure (Figure 6b). These results reveal the genetic signature of functional interaction (epistasis for fitness) between two physically unlinked genes in the same biochemical pathway, providing strong evidence that *gch1 CNP* results either directly or indirectly from antifolate selection.

In Thailand, antifolate drugs were abandoned as first line therapy ∼25 years ago, and are rarely used in neighboring Burma [24], although low levels of indirect selection may result from use of a related antifolate drug (Cotrimoxazole) used for treatment of bacterial infections [47]. Because reassortment and recombination during meiosis breaks down LD between genes, there must be strong selection favoring association of *gch1 CNP* and *dhfr-164L* in the absence of sustained antifolate selection. Hence, these data are consistent with the hypothesis that *gch1* CNP compensates for reduced efficiency of *dhfr-164L* [12]. Selection could increase *gch1* copy number within infections carrying *dhfr-164L*. Alternatively, recombinants bearing *dhfr-164L*/wild-type *gch1* or *dhfr-164I/gch1 CNP* may suffer fitness costs and fail to establish infections. Compensatory evolution involving amplification of initiator tRNA genes has previously been observed during laboratory evolution of *Salmonella*. In this case, amplification mitigates fitness costs of point mutations conferring resistance to deformylase inhibitors [48]. The associations observed between *dhfr-164L* and elevated *gch1* copy number in *Plasmodium* are all the more remarkable, because they are maintained in the face of recombination. The strength of selection required to maintain multilocus allelic combinations depends on levels of inbreeding, because this determines the rate at which recombination breaks up such combinations. In the Thai population ∼40% of infections contained multiple parasite clones, consistent with high levels of inbreeding (*f* = 0.6–0.90 [31]. Therefore, selection must be sufficiently strong to overcome breakdown of favorable allele combinations in 10–40% of the parasite population during each generation. LD is rarely observed between unlinked genes in recombining organisms: it is possible in this situation because outcrossing is rare and selection is strong.

### *Gch1* Gene Dosage Affects Gene Expression

**Figure 7:**
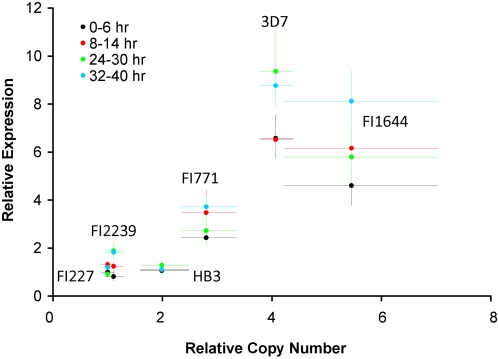
Correlation between *gch1* CNP and expression for 6 parasite isolates. We measured gene expression and copy number at 4 different time points in the asexual cycle. Error bars indicate 95% CI for estimates of both CNP and expression. Details of the real-time PCR assay are shown in Table S2. Linear regression summary statistics (slope, r^2^, F, p-value) for the four time points were as follows: 0–6 hrs: 0.63, 0.71, 10.36, 0.032, 8–14 hrs: 0.649, 0.87, 25.89, 0.007, 24–30 hrs: 0.419, 0.621, 6.54, 0.063, 32–40 hrs: 0.46, 0.85, 22.01, 0.009).

The data presented provides strong evidence that *gch1* CNP is adaptive and associated with antifolate drug selection. An important assumption made is that gene dosage is reflected in increased transcription of amplified genes. To test this assumption directly, we grew six parasites in the laboratory that vary in *gch1* copy number, and harvested mRNA at 4 different time periods in the asexual cycle. We observe strong correlations between copy number and expression in all stages of the cycle validating this assumption (Figure 7). Hence, increased expression of *gch1* has the potential to increase flux in the folate pathway. However, detailed dissection of protein flux through the pathway will be required to fully understand the impact of copy number of the dynamics of folate biosynthesis in *Plasmodium*.

### Complexity of Antifolate Evolution

The evolution of resistance to antifolate drugs has been assumed to have a simple genetic basis, because a small number of point mutations are involved and resistance can be selected in the laboratory [49]. However, molecular genetic data from regions flanking *dhfr* demonstrate surprisingly few origins and intercontinental movement of high level resistance alleles [*31*],[*50*]. Furthermore, while parasites carrying the three mutations in *dhfr* are now widespread in Africa, the *I164L* mutation, which signals the end of the useful life of pyrimethamine-sulfadoxine treatment, has not spread on this continent. Our results demonstrate that adaptation to antifolate selection is more complex than has previously been recognized, with involvement of three different components of the folate pathway (*dhfr*, *dhps* and *gch1*). This result parallels recent identification of multiple selected genes within metabolic pathways involved in skin pigmentation, hair and exocrine development, and lassa fever susceptibility in humans [*51*]. Hence, involvement of *gch1* CNP is consistent with a multilocus model for antifolate resistance evolution.

### The Nature of Selection on *gch1* CNP

The precise manner in which antifolate treatment selects for increased copy number at *gch1* remains unclear. There are two possible explanations. First, *gch1* amplification may be directly selected by antifolate treatment. In this case parasites bearing multiple copies of *gch1* might be expected to show higher levels of resistance than those carrying a single copy. We have not tested this hypothesis, which we believe is unlikely. If this were the case then we would not expect to see associations with *dhfr* in the absence of drug selection. Second, as envisaged by Kidgell et al [12], *gch1* amplification may compensate for reduced efficacy of *dhfr* and/or *dhps* enzymes bearing resistance mutations downstream in the biosynthesis pathway. Kidgell et al [12] favored compensation for fitness effects of mutations in *dhps*. However, the associations we observe between *dhfr-164* and *gch1* CNP argues strongly for involvement with *dhfr*. Ultimately, manipulation of *gch1* copy number will be required to determine the phenotypic impact of gene amplification. We caution that the effects of copy number manipulation may be strongly dependent on parasite genetic background. Hence transfection experiments designed to better understand *gch1* amplification will need to carefully consider background mutations present in *dhfr* and *dhps* and measure fitness in both the presence and absence of drug treatment.

### Screening for CNP Provides a Fast Track to Locating Genes that Underlie Adaptation

There are now six genes known that are involved in adaptation to drug treatment in *P. falciparum*. These include *pfcrt* (Chloroquine/quinine resistance), *dhfr* (pyrimethamine resistance), *dhps* (sulfadoxine resistance), *pfmdr1* (resistance to multiple drugs), mtDNA cytochrome b (atovaquone resistance) [52] and *gch1*. CNP is involved in two (*pfmdr1* and *gch1*) of these six genes (33%). We suggest that high frequency CNPs will be enriched for genes involved in adaptation, as is the case for high frequency derived SNPs [51]. Such CNPs can easily be located by cGH surveys of different parasite populations. The observation that CNP is involved in resistance to drug treatment in other pathogenic protozoa, bacteria, and cancers [*53*]–[*55*], provides further empirical evidence that CNP may be a common evolutionary response to strong selection. There is currently a strong community-wide effort to generate a high density SNP map for *P. falciparum* to help identify the genetic determinants of drug resistance and virulence by genome wide association [17]. Such SNP based screens may succeed in detecting functional CNPs. However, SNP maps in humans tend to be sparse in regions of CNP and LD between CNP and flanking SNPs is often minimal, so it is much better to determine CNP directly [1],[56]. Fortunately, this is now relatively easy using either microarray or “next generation” sequence technology. We suggest that the combination of direct assessment of CNP with dense genome-wide SNP data [6] is likely to provide a particularly powerful approach to understand the genetic determinants of adaptation to drug treatment and other selective forces in malaria parasites and other pathogens.

## Supporting Information

Figure S1 Sequences of breakpoint junctions.
(0.03 MB DOC)

Table S1 Microsatellite oligos on chromosome 12.
(0.03 MB DOC)

Table S2 Real-time PCR assays.
(0.03 MB DOC)

Table S3 Breakpoint specific PCR assays.
(0.03 MB DOC)

Table S4 Synonymous SNPs genotyped by Illumina BeadXpress.
(0.08 MB DOC)

Table S5 *Dhfr* and *dhps* alleles in Thai and Laos parasite populations.
(0.03 MB DOC)

Table S6 Dataset: SNPs, microsatellites, and real-time data.
(0.12 MB CSV)
