# Supplementary material for: Adaptive Copy Number Evolution in Malaria Parasites
Source: PLoS Genet. 2008 Oct 31;4(10):e1000243. doi: 10.1371/journal.pgen.1000243 (PMC2570623; doi:10.1371/journal.pgen.1000243)
Supplement: Figure S1 — Sequences of breakpoint junctions. (0.03 MB DOC) [file pgen.1000243.s001.doc]

**Supplementary Figure 1**

**Sequencing of chromosomal breakpoints**.

We amplified across chromosomal breakpoints using the PCR primers listed (Table S3). Alignment of breakpoint junction sequences is shown with 3D7. For each of the amplicons characterized the junction sequence is shown (top line). This is aligned with the matching 3D7 sequence on each side of the junction site (lines 2 and 3 for each amplicon). Brackets indicate the position of the junction sites. Dots indicate matches, while bases are shown when there are mismatches with the breakpoint junction sequences.

Isolate

1.7kb amplicon

3D7 ACAATAGTATTATATAATATATATGTATATGTATATGTATATATATTT(TA)TTATACATTTAACTTAAAAATAATATTTTTATATTATTTATACATGTGT

3D7(5') CTTT.TT.T..T.T.TT.T.T.T..GTA..C....AA.......CCAA(17).................................................

3D7(3') ................................................(21).GCATTTAAA.GAC.TTTC..T.A.AA.A.....A.A.A.A.ACATAAA

2.2kb amplicon

M48 ACAATAGTATTATATAATATATATGTATATGTATATGTATATATATTT(TA)TTATTTATGGATGTATAGTTTACTAAAATGAATTAGATTCTATAATATT

3D7(5') .T.TATA...ATAT..T..A...ATA.A..AA.A.CAGTA.C.C..A.(13).................................................

3D7(3') ................................................(21).GCA..TAAA.GACT.TTCA.TAA...TAT.TA..A..AAA.AC...AA

7.3kb amplicon

M222 CAAAACATGCTTCTACATATTTATATATATCAAAATTACAAATTACGT(AA)CATATACATATATATATATATATATATATATATATATATATATTTATTT

3D7(5') T.T..T.ATTG.G..T.....ATA...GT.ACCC.AAT.T.TC.TAAT(35).................................................

3D7(3') ................................................(8 )T.....T......................TATA.ACACATACA.A.CA.

8.7kb amplicon

M205 TATATACATGTTGTTATATTTAACAAATATTAATATATAACAATTTTG(G(T)6)CTCTGTTTCCTTTTTTAGTCACAATTTACTAATCAAAAGCCTATTACGT

3D7(5') ATG.ACA.ACG.ACATATAA..TAC.....A..ACC.CCCT.C.A.AT(2 ).................................................

3D7(3') ................................................(7 )GAT.T.GATT......GTGTGTTT...TT.TT..TTTTTTT.CG.TT.G
