# Supplementary material for: Adaptive Copy Number Evolution in Malaria Parasites
Source: PLoS Genet. 2008 Oct 31;4(10):e1000243. doi: 10.1371/journal.pgen.1000243 (PMC2570623; doi:10.1371/journal.pgen.1000243)
Supplement: Table S4 — Synonymous SNPs genotyped by Illumina BeadXpress. (0.08 MB DOC) [file pgen.1000243.s005.doc]

**Supplementary Table S4. Synonymous SNPs genotyped in Thai and Loas samples.** The alternative states at each SNP are shown in square brackets with 50bp sequence on either side (see column "Sequence"). SE Asian isolates showing alternative SNP states in existing genome sequence data at each of these SNPs are shown. These were run as controls. At 5 loci the expected SNP was not found: these are marked with *. These probably represent incorrect base calls in genome sequence data.
