# Supplementary material for: Adaptive Copy Number Evolution in Malaria Parasites
Source: PLoS Genet. 2008 Oct 31;4(10):e1000243. doi: 10.1371/journal.pgen.1000243 (PMC2570623; doi:10.1371/journal.pgen.1000243)
Supplement: Supplementary file 3 [file pgen.1000243.s002.doc]

Supplementary table S1. Microsatellite oligos used to genotype chr 12.

Position		Forward oligo	Reverse oligo	Product size_3D7
198899		AATTTTTCTCTACAAAAGGA	TATAGCACATCCCGATATAA	128
349302		ACAAACAAATTCACAAGAAA	TACAAATAGAACACCAAAAA	144
498441		AACAAATTGAAACTGACCTA	TGGCAAGTAGACTCTTTTTA	229
698587		AAATAAAAGTTTCACCATGT	AGGAGACCATGTTTTGAC	147
800675		AAAAACAAAAAGACCTACAT	TTTTAAGGTTGTGTATAATCTG	98
850501		TATAAATGTGCTATGCAAAA	GCAGATGTAGTCATAAGCAT	167
900432		AACTGTCCGTTTCATCTT	CCCATTTGTGATATATTTTT	160
917258	*	CATTAATAGTATAATTAGCTTTATGTT	TTTTCAATCAAATTACTTTACA	127
925760	*	AGACAAAAATATGATAACGTGT	TAAAGTTGAAAAATAACAACAG	139
938880	*	CATCTGATAATGTTATACAAAAA	CATCTATATGAACAAGACAAAA	171
947562		TGGTGAGATAAAACTGAGAT	TTTACATTTTATGGTTCTGTT	135
966803	*	ACTTTGGTAAACCTAAAAATTA	GTACATTATATAAACGCACAGA	129
970980	*	TTCTAAAATGAATTGTACTTGA	CAAAACAATATTTAACAACACA	221
972580	*	AATATATTTATTAAGACTACGAAAAA	GGAAAAACATAGAAAAATTATG	195
974240	*	TTGGTAATCTATAAATATATATCCA	CAGTCCATTAAATGAAAAATA	164
975700	*	TCGCTAACAATTTTGATATAC	TTTATACCCCTATGAATCAA	238
976160	*	TATCAAAATTACAAATTACG	AAGAAATCACAACTTTTCAA	179
976390	*	TTGAAAAGTTGTGATTTCTT	TGTGATTATCATGACATTGTT	206
977000	*	TAATTCTTGAAAACTTGCTT	GTGTGTATTTTGGTATGGAG	296
981230	*	TTAGTATACACATTTCCTTCTATT	TTCTTAATTAGCTCTTTCAAAT	239
983610	*	AATAAGATATGTTTGCAGGA	TTTCCAACAGAACATTTAAC	235
984557	*	TGAACGTTATTACTGATAAAAG	GTTTCACCATTCTTAGTAAGTT	233
1001332		TTTTTATCAAAATGGATGTA	TATTGACAACACGGAATAAT	141
1010454	*	TTTTAATTTTCACATAGGACA	TATTTATATTTTCCCACTTTTT	231
1029280	*	ATATCACCATGTAATAAACAAT	ACGACAAAATAATAACAAAAC	221
1044051	*	GGAAAGAAAACATGTAGAATTA	AATAAAAACTGTGTAATTAGGT	143
1097870		TATCCATAAATAATGCATCC	TTCTTTTCTCACTTATTTCG	205
1148032		GGAATAAGAATGCTCATAAA	TGTAATCATAGGACAGAAAAA	134
1300046		AGTTATATCCCCAAAAGATT	AATCAGTATATCCCATATAAAAA	254
1450553		ACAGTTAGGAGAGACAAACA	AAAAGCAATGTTAAAATGAG	244
1600878		TCAAGATAACTCTTCCTCAA	ATGTAATTGTGTTTGGTTTT	305
1745963		TGTTTATGAGATGATGTGTG	GGGTAATAATATATAAATCCTTGT	240
1898967		AATTGACATACACAAAAAGG	CGTCATCTTTCATTTCTTT	220

* The M13 tailed labelling method was used to score these loci. The M13 oligo (tgtaaaacgacggccagt) was addred to the 5' of the forward oligo, while a 7 bp tail (gtttctt) was added to the reverse oligo. An M13 oligo with a 5' fluorescent label was then added to each reaction to label PCR products.
