# Supplementary material for: Adaptive Copy Number Evolution in Malaria Parasites
Source: PLoS Genet. 2008 Oct 31;4(10):e1000243. doi: 10.1371/journal.pgen.1000243 (PMC2570623; doi:10.1371/journal.pgen.1000243)
Supplement: Supplementary file 4 [file pgen.1000243.s003.doc]

Supplementary table S2. Real time PCR assays on chr 12. The endogenous control is a VIC labelled MGB probe. Other probes are 6FAM labelled MGB probes. We run single plex assays in quadruplicate and measure relative copy number using the $\Delta\Delta C_T$ method

Position	Probe sequence	Forward Primer	Reverse Primer	Gene
Test genes				
966681	AATTACATTGACAACATTCC	CCTAAATCAAAATGTTGCGGAAA	ACCCTTTACATATCCAAGCAACAAA	PFL1135c: hypothetical protein
969971	ACGTGTGCCCTTTAT	CAAGTCACAATTTACTAATCAAAAGCCTAT	ACCATTCCTGCAATCATTCCA	PFL1140w: hypothetical protein
971971	CTGTTTGGAATAACCC	TGGAATTGTTGGAGGTGCTCTT	TGCATATAAACCCGAACCACATC	PFL1145w: hypothetical protein
972951	CGTACACATTCACCGTTCA	AAACCAACAGTTGTCAAAAGGAGAT	CATTGCACACCCTGAAATTTTAGA	PFL1150c: ribosomal protein L24, putative
975258	CTTGAAAATTTAGATAACCCGA	CCTTTTGAAGGTACATGTGATATTGAGT	GCGTTACAAATATCGTTAGTTAAATCTTCT	PFL1155w: GTP cyclohydrolase I
977841	ACCGTTCGACTATATTTTA	CGACGACGTGTCACCATATAGATAA	CAAGTTGTTTCAGATTTGACGAACTT	PFL1160c: hypothetical protein
981321	ACGACAAGGTATCCC	CAAGTTGTTTCAGATTTGACGAACTT	CATCAAAGCAAAATTCAGGATTCTT	PFL1165w: hypothetical protein
989420	CCTAAAAAAGAGGAAGCTACT	GATGATGGACAAATAGATGAAACATATGA	TTGCTGCTCCACTTGTTGATG	PFL1170w: polyadenylate-binding protein
996220	TATAATCCTTCACACAATATGCT	AAAAATTTGACCTTACGAGTTCATGA	GTTAAACCATGGGACATTACAATAAACA	PFL1175w: hypothetical protein
Endogenous control	CCAATAATTTCTGCCATACTA	TTAGATTTTCAAGCGAGACGTTTAAA	CCTTCCTACGGCTAAACCTGAAC	PFL0770w: Seryl-T synthetase
