# Supplementary material for: Adaptive Copy Number Evolution in Malaria Parasites
Source: PLoS Genet. 2008 Oct 31;4(10):e1000243. doi: 10.1371/journal.pgen.1000243 (PMC2570623; doi:10.1371/journal.pgen.1000243)
Supplement: Supplementary file 5 [file pgen.1000243.s004.doc]

Supplementary table S3. Breakpoint specific PCR assays for amplicons in Thailand. These primer pairs amplify across unique junctions that are formed during tandem duplication of chromosomal segments containing *gch1* , but produce no product in a parasite samples that contains a single copy of *gch1* . The location of the 5' and 3' breakpoints in the genome sequence of parasite 3D7 are shown.

Amplicon size	forward and reverse primers	5' break[d]	3' break[d]	Breakpoint sequence	Gene content[e]
1.8kb [a]	TGATTCATAGGGGTATAAAA<br>CAGTCCATTAAATGAAAAAT	974251	976019	$(TA)_n$	GTP cyclohydrolase I (PFL1155w)
2.2kb[b]	TGATTCATAGGGGTATAAAA<br>AGGAATAAAGAGTAAACAAAAC	973801	976019	$(TA)_n$	GTP cyclohydrolase I (PFL1155w)
8.7kb	TTCTAATATGGAAAATCATTC<br>CGTAATAGGCTTTTGATTAG	969921	978641	$(GTTTTTT)_n$	HP (PFL1140w), HP (PFL1145w), ribosomal protein L24 (PFL1150c), GTP cyclohydrolase I (PFL1155w), HP (PFL1160c)
7.2kb	TGATTCATAGGGGTATAAAA<br>TCACCATGCTTATCTAAGTT	968861	976141	A_n	HP (PFL1140w), HP (PFL1145w), ribosomal protein L24 (PFL1150c), GTP cyclohydrolase I (PFL1155w)
>11kb [c]	nd				

[a] Identical chromsomomal breakpoints were found in the genome sequence strain 3D7.

[b] One of two samples with amplified *gch1* from Laos also amplified with the same breakpoint specific primer set

[c] A single sample from both Thailand and Laos had amplicons that were larger than the span of our realtime PCR assays on the 5' side. Breakpoints for these samples were not characterised. However, the 3' boundary fell in the same location (977841-981321bp) in both Thai and Laos samples suggesting that these amplicons may be the same.

[d] The breakpoints were intergenic in repetitive sequences. As a consequence the precise position of the breakpoints cannot be determined.

[e] HP = Hypothetical protein
