# Supplementary material for: Adaptive Copy Number Evolution in Malaria Parasites
Source: PLoS Genet. 2008 Oct 31;4(10):e1000243. doi: 10.1371/journal.pgen.1000243 (PMC2570623; doi:10.1371/journal.pgen.1000243)
Supplement: Supplementary file 6 [file pgen.1000243.s006.doc]

Supplementary Table S5. Frequencies of *dhfr* and *dhps* alleles in Thailand and Laos parasite populations. Five key amino acids at each locus were genotyped by SNaPshot. The 5 amino acid codes designate alleles. These correspond to amino acids 16, 51, 59, 108 and 164 for dhfr and to amino acids 436, 437, 540, 581 and 613 for dhps. Wild-type amino acid states are shown in plain text while amino acid changes known to confer resistance are shown in bold. One unusual dhfr allele (marked with *) containing the 59R but 108S was found in 5 Lao individuals. This allele has not be confirmed by sequencing.

***dhfr* allele**	**Thai (n=139)**	**Laos (n=149)**	***dhps* allele**	**Thai (n=137)**	**Laos (n=148)**
ANCSI	---	0.23	SAKAA	---	0.83
ANC**N**I	---	0.03	AAKAA	---	0.01
*AN**R**SI	---	0.03	S**G**KAA	---	0.07
AN**RN**I	0.04	0.34	AA**E**AA	---	0.01
**V**NC**T**I	---	0.01	A**G**KAA	---	0.01
A**IRN**I	0.16	0.35	S**G**KGA	0.08	---
AN**RNL**	0.09	---	A**GE**AA	0.34	0.04
A**IRNL**	0.71	---	S**GEG**A	0.58	---
			**FGE**A**S**	---	0.02
